# Tcf7l1 promotes transcription of Kruppel-like factor 4 during *Xenopus* embryogenesis

**DOI:** 10.7555/JBR.32.20170056

**Published:** 2017-11-30

**Authors:** Qing Cao, Yan Shen, Wei Zheng, Hao Liu, Chen Liu

**Affiliations:** 1. College of Medicine, Henan University of Science and Technology, Luoyang, Henan 471023, China; 2. Department of Developmental Genetics, Nanjing Medical University, Nanjing, Jiangsu 211166, China.

**Keywords:** Kruppel-like factor 4 (Klf4), Tcf7l1, transcription regulation, *Xenopus* laevis

## Abstract

Kruppel-like factor 4 (Klf4) is a zinc finger transcription factor and plays crucial roles in *Xenopus* embryogenesis. However, its regulation during embryogenesis is still unclear. Here, we report that Tcf7l1, a key downstream transducer of the Wnt signaling pathway, could promote *Klf4* transcription and stimulate *Klf4* promoter activity in early *Xenopus* embryos. Furthermore, cycloheximide treatment showed a direct effect on *Klf4* transcription facilitated by Tcf7l1. Moreover, the dominant negative form of Tcf7l1 (dnTcf7l1), which lacks *N*-terminus of the β-catenin binding motif, could still activate *Klf4* transcription, suggesting that this regulation is Wnt/β-catenin independent. Taken together, our results demonstrate that Tcf7l1 lies upstream of Klf4 to maintain its expression level during *Xenopus* embryogenesis.

## Introduction

Klf4, a zinc finger containing transcription factor of the Kruppel-like factor family, plays critical roles in stem cell biology and early embryogenesis. As a pluripotency factor, Klf4 is required for maintaining pluripotency, self-renewal of embryonic stem (ES) cells and reprogramming of differentiated cells^[1^–^2]^. In fact, knockdown of *Klf4* results in the differentiation of ES cells^[3^–^4]^. During *Xenopus* embryogenesis, *Klf4* is expressed maternally and zygotically^[5]^, which is vital for germ layer formation and body axis patterning. Overexpression of *Klf4* promotes neural precursor and endoderm formation, whereas knockdown of *Klf4* leads to the failure of three germ layer differentiation^[5^–^6]^. Due to its important functions in cell fate determination in both ES cells and early embryos, Klf4 should be maintained at a correct level so that cell differentiation could continue properly. A few attempts so far have been done on the transcription regulation mechanism of Klf4 in ES cells, some cancer cells and adult cells^[7^–^9]^. It has been reported that P53^[10]^, CDX2^[11]^, histone modification^[12]^, microRNA^[13^–^15]^, BMPs and TGFβs^[16^–^17]^ could regulate *Klf4* expression. Besides the mechanism described above, Klf4 could upregulate its own transcription by binding to its promoter^[1^,^18]^. Although the previous studies have provided some clues of the regulation mechanism of *Klf4*, there is still little information available in literature about the regulation of *Klf4* transcription during early embryogenesis.

Tcf7l1 (also known as Tcf3) is a key downstream transducer of the Wnt signaling pathway, which is required for early body axis specification and mesoderm induction^[19^–^23]^. It is maternally expressed in *Xenopus* embryos and plays dual functions (repressor or activator) in regulating gene expression^[24]^. Depletion of Tcf7l1 showed headless phenotype in both zebrafish and *Xenopus* embryos^[25^–^26]^. Furthermore, Tcf7l1 is reported as an integral component of the regulatory circuitry of ES cells^[27]^. In ES cells, Klf4 and a few other transcription factors, e.g. Oct4, Sox2, Myc and Nanog, etc., comprise a core genetic circuitry to maintain pluripotency and self-renewal of ES cells^[1]^. Recently, it has been demonstrated that Tcf7l1 is involved in regulation of *Oct4*, *Sox2*, *Nanog* and *Klf4* transcription in ES cells^[27^–^28]^. Embryogenesis is a process of cell differentiation by loss of pluripotency and the pluripotency factors play important roles in this process^[6]^. Based on the research in ES cells described above, we aim to know whether Tcf7l1 likewise regulates *Klf4* transcription *in vivo*.

In this paper, we aimed to investigate the effect of Tcf7l1 on *Klf4* transcription during *Xenopus* early embryogenesis through gain of function and loss of function analyses. First, we found that *Klf4* expression is strongly upregulated in response to overexpression of *Tcf7l1* both in whole embryos and animal caps. Secondly, knockdown of Tcf7l1 leads to reduction of *Klf4* expression. Thirdly, dual-luciferase reporter assay showed that *Klf4* promoter activity is dramatically stimulated by Tcf7l1. Finally, we demonstrated that the transcription of *Klf4* promoted by Tcf7l1 is a direct effect and seems to be Wnt/β-catenin independent. In summary, our results suggest that Tcf7l1 lies upstream of Klf4 and is required for *Klf4* transcription during *Xenopus* embryogenesis.

## Material and methods

### Embryos and explants

*Xenopus laevis* embryos were obtained, cultured and staged as previously described^[5]^. Animal caps were excised from stage 8 embryos and collected at stage 10.5. To block protein translation, embryos were incubated in 0.1 × MBSH (1×MBSH: 88 mmol/L NaCl, 2.4 mmol/L NaHCO_3_, 1 mmol/L KCl, 0.82 mmol/L MgSO_4_, 0.41 mmol/L CaCl_2_, 0.33 mmol/L Ca(NO_3_)_2_, 10 mmol/L HEPES, pH-7.4) containing cycloheximide (CHX) at 25 μg/mL from stage 7 to stage 10.5 (gastrulation).

### ***In vitro*** transcription, morpholino oligonucleotides and microinjection

To prepare mRNAs for microinjection, plasmids pCS2+ Tcf7l1 and pCS2+ dnTcf7l1 were linearized by *Not*I, transcribed with Sp6 mMessage mMachine kits (Ambion) and cleaned up with RNeasy Kit (Qiagen). An antisense morpholino oligonucleotide (MO), Tcf7l1 MO: 5′-CGCCGCTGTTTAGTTGAGGCATGA-3′, was used to knock down *Xenopus laevis* Tcf7l1 as previously reported^[19]^. The standard control MO (ctrlMO) 5′-CCTCTTACCTCAGTTACAATTTATA-3′ against the human β*-globin* gene was used as a control. All MOs were purchased from GeneTools. Injected doses of mRNA or MOs are described in the text.

### Whole-mount ***in situ ***hybridization

A digoxigenin-labeled probe of Klf4 was prepared by digesting plasmids pCS2+ Klf4 with *Cla*I, transcribing with T7 RNA polymerase (Fermentas) and cleaned up with RNeasy Kit (Qiagen). *Xenopus laevis* embryos were fixed in 1×HEMFA (0.1 mmol/L HEPES, 2 mmol/L EGTA, 1 mmol/L MgSO_4_, 4% formaldehyde, pH7.4) for 2 hours and dehydrated twice in absolute ethanol for 10 minutes. Whole-mount *in situ* hybridization was carried out according to standard protocols of Harland (1991)^[29]^ except for using BM purple (Roche) instead of NBT/BCIP for chromogenic reaction.

### cDNA synthesis and quantitative RT-PCR

Total RNA was extracted from embryos or animal caps with Trizol (Qiagen). First strand cDNA was synthesized from 2 μg total RNA with RevertAid^TM^ First Strand cDNA Synthesis kit (Fermentas). cDNA was used at a dilution of 1:40 for Quantitative RT-PCR (qPCR). Amplification parameters were as follows: one cycle of predenaturation at 95°C for 10 senconds, followed by 40 cycles of denaturation at 95°C for 5 seconds, annealing and extension at 60°C for 31 seconds and an additional cycle for the melting curve. Crosspoints were calculated using ABI 7300 system SDS software and normalized according to the expression level of ODC (ornithine decarboxylase) included in each run. Final results were presented as histograms with relative units. qPCR primers are listed below: *Klf4* (F): 5′-GAGAGGTGGAGGGAAGATCCA-3′; *Klf4* (R): 5′-CCAAACCATCATAAGCACGAGAC-3′; *ODC* (F): 5′-CAAAGCTTGTTCTACGCATAGCA-3′; *ODC*(R): 5′- GGTGGCACCAAATTTCACACT-3′.

### Construction of ***Xenopus Klf4*** promoter reporter plasmid

To further study the transcription mechanism, we need to isolate the promoter sequence of *Klf4*. Because the genomic data for *Xenopus laevis Klf4* gene is not available, we chose *Xenopus tropicalis* to do such an experiment, which is a close relative of *Xenopus laevis* and whose genomic DNA is completely sequenced. It has been reported that genes and regulatory mechanism are conserved between *Xenopus laevis* and *Xenopus tropicalis*. Since *Xenopus laevis* Klf4 and *Xenopus tropicalis* Klf4 have 94% similarities^[5]^, we used *Xenopus tropicalis Klf4* promoter sequence to carry out luciferase assay. The DNA of Klf4 promoter region −2144/+70 (transcription start as+1) was amplified from a library of *Xenopus tropicalis* genomic DNA and was subcloned to pGL3-basic to generate Klf4Luc (−2144/+70).

### Luciferase assay

To perform luciferase assays on *Xenopus* embryos, promoter reporter plasmid DNAs and mRNAs or MOs were co-injected into equatorial region of all blastomeres at 2 or 4-cell stage. Embryos were collected at the gastrula stage, and homogenized in 1×passive lysis buffer (Promega) by using tips. Homogenates were incubated at room temperature for 10 minutes and centrifuged at 12,000 r/min for 10 minutes at 4°C. Then, the supernatants were transferred to fresh tubes and used for luciferase assay. Luciferase activity was measured with 20 μL lysate of each sample and 100 μL of luciferase substrate (Promega) using a Lumat LB 9507 luminometer (Berthold). Luciferase activities are presented as diagrams with relative units compared to the value of plasmid DNA injection alone. Each reporter assay was carried out at least 4 times independently.

### Statistical analysis

Statistical analysis was calculated using Graphpad Prism5 software. Data were presented as mean±SD. Differences between groups were determined by Student's *t*-test with significance at *P*≤0.05 (* indicate *P*<0.05 and ** indicate *P*<0.01).

## Results

### Tcf7l1 promotes ***Klf4*** transcription in early*** Xenopus*** embryos

**Fig.1:**
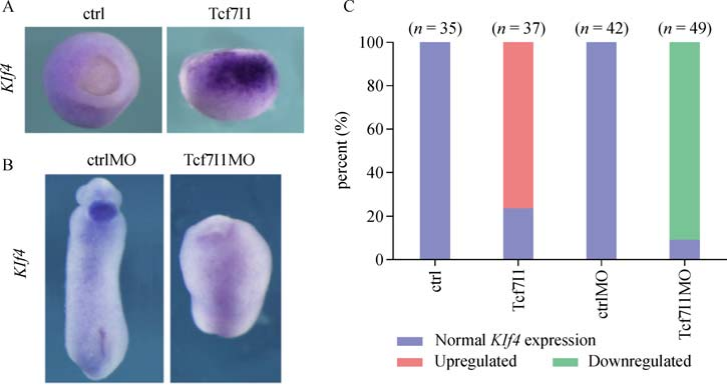
***Klf4*** expression is upregulated by Tcf7l1. A: 300 pg of *Tcf7l1* mRNAwas injected into 2 or 4-cell stage embryos. Then, the embryos were collected at stage 10.5 for whole-mount *in situ* hybridization by using *Klf4* probe. B: 2 or 4-cell stage embryos were injected with 40 ng ctrlMO or Tcf7l1MO and were collected at stage 28 to test Klf4 expression. C: Quantification of embryos with normal or altered gene expression observed in A and B in two experiments.

First, the effect of Tcf7l1 on *Klf4* transcription was examined through whole-mount *in situ* hybridization in early *Xenopus* embryos by loss of function and gain of function. During gastrulation, the expression level of Klf4 was very low in uninjected control embryos, while *Klf4* transcription level was dramatically upregulated in the animal pole and the lateral region in embryos injected with *Tcf7l1* mRNA (***Fig. 1A***, ***C***). Conversely, when endogenous Tcf7l1 was knocked down by injecting Tcf7l1 MO, which was described previously^[19]^, *Klf4 *transcription was eventually diminished. At the tailbud stage, *Klf4* was specifically expressed in the cement gland. Embryos injected with Tcf7l1 MO showed a headless phenotype with no cement gland formation, and *Klf4* expression was severely downregulated (***Fig. 1B***,*** C***). Our results showed that Tcf7l1 dramatically activated *Klf4* transcription. Loss of Tcf7l1 function leads to downregulation of *Klf4* in *Xenopus* embryos.

**Fig.2:**
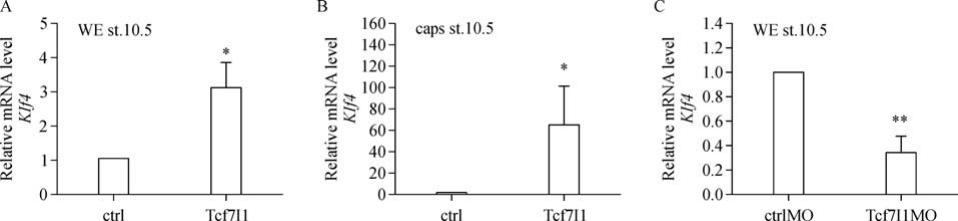
Tcf7l1 promotes ***Klf4*** transcription in both whole embryos and animal caps. A: 300 pg of *Tcf7l1* mRNA was injected into 4-cell stage embryos, control and injected embryos were collected at stage 10.5. B: For animal cap assay, animal caps were cut at stage 8 and were collected until control embryos reached stage 10.5. C: *ctrlMO* and *Tcf7l1* MO were injected at 40 ng, respectively, and embryos were collected at stage 10.5 for qPCR. Data were presented as mean±SD. Differences between groups were determined by Student's *t*-test. * indicates *P* < 0.05. **indicates *P* < 0.01. qPCR was carried out in three experiments.

Second, in order to confirm the phenotype, *Klf4* expression in the whole embryos and animal caps was tested by using qPCR. Overexpression of *Tcf7l1* led to significant upregulation of *Klf4* in both whole embryos and animal caps (***Fig. 2A***,*** B***). In contrast, *Klf4* expression was dramatically reduced in embryos with knockdown of Tcf7l1 (***Fig. 2C***). The qPCR results confirmed that *Klf4* expression was indeed regulated by Tcf7l1.

### Tcf7l1 stimulates ***Klf4*** promoter activity

**Fig.3:**
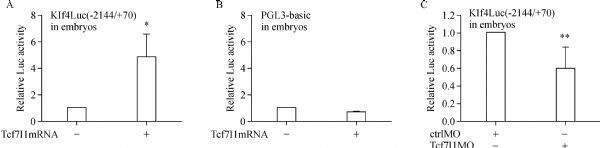
*Tcf7l1* stimulates Klf4Luc(−2144/ + 70) activity. A and B: 40 pg of Klf4Luc(-2144/ + 70) or PGL3-basic plasmid was injected alone or together with 300 pg Tcf7l1 mRNA, embryos were collected at stage 10.5 for luciferase assay. C: 40 pg Klf4Luc(-2144/ + 70) was co-injected with 20 ng of ctrlMO or Tcf7l1MO, respectively, embryos were collected at stage 10.5 for luciferase assay. Luciferase reporter assay was carried out at 5 (A, B) or 4 (C) independent times. Data were presented as mean±SD. Differences between groups were determined by Student's *t*-test. *indicates *P* < 0.05. ** indicates *P* < 0.01.

To further study the regulatory mechanism of Tcf7l1 on *Klf4* transcription, we performed luciferase reporter assay. *Xenopus tropicalis Klf4* promoter sequence −2144/+70 was subcloned into PGL3-basic vector and the luciferase reporter, Klf4Luc (−2144/+70), was constructed. In embryos, Klf4Luc (−2144/+70) was significantly stimulated by overexpression of *Tcf7l1* because the luciferase activity was much higher in embryos injected with both the reporter plasmid and *Tcf7l1* mRNA than in embryos injected with the reporter alone (***Fig. 3A***). The stimulation was specific since the vector pGL3-basic, which was used for making the reporter construct, was not stimulated by overexpression of *Tcf7l1* (***Fig. 3B***). On the contrary, when *Tcf7l1* MO was injected into the embryos, luciferase activity was decreased appreciably (***Fig. 3C***). These series of experiments clearly demonstrated that Tcf7l1 stimulates *Klf4* promoter activity.

### *Tcf7l1* promotes ***Klf4*** transcription in the absence of protein translation

**Fig.4:**
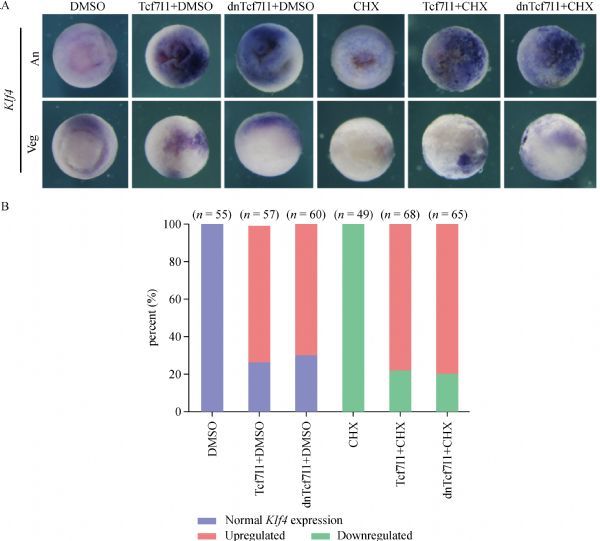
Cycloheximide (CHX) treatment shows that ***Klf4*** expression is directly upregulated by Tcf7l1. A: *Klf4* expression is shown in response to injection of *Tcf7l1* mRNA or *dnTcf7l1* mRNA, to treatment with CHX, to injection of *Tcf7l1* mRNA or *dnTcf7l1* mRNA together with CHX treatment. Embryos were orientated to animal view (An) and vegetal view (Veg). *Tcf7l1* mRNA or *dnTcf7l1* mRNAwas injected at 300 pg of each embryo. B: Quantification of embryos with normal or altered gene expression observed in three experiments.

To further examine whether Tcf7l1 regulated *Klf4* transcription directly or not, we performed CHX treatment to block protein translation. Overexpression of *Tcf7l1* with or without CHX both showed dramatic upregulation of *Klf4* expression (***Fig. 4A***,*** B***). The results suggested that even in the absence of protein translation, Tcf7l1 was still able to promote *Klf4* transcription. Furthermore, overexpression of a dominant negative form of Tcf7l1(dnTcf7l1), which does not contain the β-catenin binding motif, showed a similar effect on *Klf4* transcription suggesting that without β-catenin, Tcf7l1could still activate *Klf4* expression.

## Discussion

Klf4 plays important roles not only in maintaining pluripotency of embryonic stem cell, but also in early embryogenesis. In our previous work, Klf4 could induce endoderm differentiation through regulating Nodal/activin signaling pathway^[5]^. Zhang *et al*. and Evans *et al*. have reported that Klf4 could interact with β-catenin and Tcf7l2 (formerly named TCF4) in colorectal cancer cells and inhibit body axis formation in *Xenopus* embryos^[30^–^31]^. Feedback loops among different signals are frequently present during embryogenesis and are essential for maintaining the homeostasis of signals, so that they can function in the correct time, space and at correct levels. Since Klf4 has the ability to regulate Wnt and Nodal/activin signals^[5^,^30^–^31]^, which play key roles in germ layer differentiation and body axis formation, we aim to know whether Nodal/activin or Wnt pathways could regulate *Klf4* transcription. So we examined the regulatory effect of the key nuclear signal transducers in Nodal/activin or Wnt signaling, especially FAST1 and TCF/LEF. Our results showed that overexpression of *FAST1* in embryos did not generate significant effect on *Klf4* transcription, as revealed by whole-mount *in situ* hybridization (***Supplementary Fig. 1***, available online). Nevertheless, *Klf4* transcription level was augmented strongly in embryos injected with *Tcf7l1* mRNA.

In the present study, we focus on the regulatory effect of Tcf7l1 on *Klf4* expression. We found that *Klf4* expression is dramatically decreased in embryos with Tcf7l1 knockdown. On the contrary, overexpression of *Tcf7l1* in whole embryos and animal caps increased *Klf4* expression significantly by both qPCR and whole-mount *in situ* hybridization. These results suggest that Tcf7l1 could promote *Klf4* transcription during *Xenopus* embryogenesis. *Xenopus* germ layer formation is a zygotic event and does not begin until mid-blastula (stage 8.5), so zygotic gene transcription has not started at stage 7. To further study the effect of Tcf7l1 on *Klf4* is direct or not, we performed CHX treatment. Activation of *Klf4* was still observed when *Tcf7l1*-injected embryos were treated with CHX form stage 7 to stage 10.5. It is suggested that activation of *Klf4* by Tcf7l1 was a direct effect and not through activation of other zygotic genes to regulate *Klf4* transcription. In addition, luciferase reporter assays showed that overexpression of *Tcf7l1* stimulates *Klf4* transcription. Based on our data, we have demonstrated that Wnt downstream transducer Tcf7l1 lies upstream of Klf4 and promotes *Klf4* transcription.

Tcf7l1 transduces Wnt signaling *via* interaction with β-catenin, but the regulatory effect seemed to be Wnt/ β-catenin independent, because overexpression of β-catenin in embryos didn't change *Klf4* expression significantly (***Supplementary Fig. 2***, available online). Moreover, a dominant-negative form of Tcf7l1 (dnTcf7l1) that does not bind β-catenin showed the similar effect on *Klf4* transcription. This suggested that Tcf7l1 could activate *Klf4* transcription and the effect seems to be Wnt/β-catenin independent. However, the details of how Klf4 is regulated by Tcf7l1 are still unclear and need to be further explored. Embryogenesis is a process regulated *via* differentiation signals and the pluripotency factors, so our studies have provided a deeper understanding of the regulation of Klf4 *via* differentiation signals during early embryogenesis.
